# Exosomes in Cancer Diagnosis and Therapy

**DOI:** 10.3390/ijms23179930

**Published:** 2022-09-01

**Authors:** Aamir Ahmad

**Affiliations:** Translational Research Institute, Academic Health System, Hamad Medical Corporation, Doha 3050, Qatar; aahmad9@hamad.qa; Tel.: +974-44390984

Cancer affects millions of people worldwide every year. In the year 2020, for which the latest global statistical data are available [1], more than 18 million cancer cases were diagnosed globally. This corresponds to 206.9 cancer diagnoses per 100,000 men and 178.1 cancer diagnoses per 100,000 women. In the United States alone, more than 1.9 million new cancer cases are expected to be diagnosed in 2022 [2]. These numbers are alarming. Of note, aggressive screening and growing awareness have contributed to the increased numbers [3]. However, it is critical to reduce the mortality, which stood at 10 million globally for the year 2020 [1] and is expected to be more than 600,000 in the United States this current year [2].

To reduce cancer-associated mortality, it is important to better understand the mechanisms that facilitate tumor growth, so that appropriate targeted therapies can be designed. Cell-to-cell communications in the close vicinity of tumor cells play an important role in creating a tumor microenvironment that is conducive to tumor growth [4]. The extracellular vesicles, exosomes, facilitate cell-to-cell communications, wherein they serve as cargos for the transport of proteins, RNA, miRNAs, non-coding RNAs, etc. [5,6]. This type of exosome-mediated transport is believed to confer survival advantages to the growing tumor, through modulation of the immune response and affecting angiogenesis, metastasis and therapy resistance. The Special Issue ‘*Exosomes in Cancer Diagnosis and Therapy*’, published as part of the section ‘*Molecular Pathology, Diagnostics, and Therapeutics*’, comprises of a total of 15 articles, including 4 original research articles and 11 review articles. The articles published in this Special Issue covered the very basics of our evolving understanding of exosomes [5], cancer progression [7], their role within the tumor microenvironment [8] and even the different available techniques for their extraction [9].

One of the earliest submissions to this Special Issue dealt with the biodistribution of exosomes [10]. Since exosomes are being investigated for their exploitation in cancer therapy, the knowledge on their in vivo distribution is necessary. This review article evaluated twenty-nine published articles to extract the available knowledge, with a focus on molecular imaging. Another article proposed milk-derived exosomes as efficient agents for cancer-targeted drug delivery [11]. The current technology is not yet developed enough to generate artificial exosomes, thus leaving physiological exosomes as the only choice, and milk happens to be an exosome-harboring biological fluid that is commercially available. Thus, harnessing exosomes from milk to deploy them in targeted therapy against cancer is promising. An advantage of using exosomes, such as those from biological fluids, as delivery vehicles for anti-cancer therapy, is their low immunogenicity. Additionally, they are highly stable in blood circulation and, moreover, they can be suitably engineered [12] to either display targeting moieties or add additional therapeutics as their cargo, leading to more effective inhibition of tumor growth. Exosomes can also modulate immunity and even cryopreserved and readily available sources, such as exosomes from cryopreserved umbilical cord blood, can be candidate materials for vaccination against cancer [13]. In particular, DC-derived exosomes have the potential to mitigate the side-effects associated with DC-based vaccinations. In the field of cancer immunotherapy, there has been considerable interest in signaling through PD-L1 [14], which might be of particular relevance to the treatment of TNBCs that have high levels of PD-L1 [15]. The work by Zhou et al. suggests targeting of PD-L1 by exosomal miR-424-5p, with concomitant generation of pro-inflammatory cytokines in the tumor microenvironment and increased apoptosis of TNBC cells MDA-MB-231 [15]. The tumorigenic potential of inflammation-associated cytokine signaling is well known [16]; therefore, its modulation by exosome cargo is of significance.

In addition to therapy, exosomes are also being sought for their role in cancer diagnosis [17]. The possible exploitation of exosomes in the diagnosis of retinoblastoma is discussed in an article by Lande et al. [18]. Exosomes are attracting significant attention for their dual role in diagnosis as well as in the therapy of different cancers, such as pituitary adenomas [19] and skin cancer [20]. Skin is the largest human organ that is constantly exposed to external stimuli. It is increasingly being realized that exosomes affect the pathogenesis of various cutaneous diseases, such as melanoma, non-melanoma skin cancer, chronic cutaneous inflammatory conditions and even skin autoimmune diseases [20]. An article published in this Special Issue focuses on the rare RNA species carried by exosomes that hold promise for early cancer diagnosis, leading to better patient outcomes [21]. While non-coding RNAs, such as miRNAs, lncRNAs and circRNAs, have been a major focus of interest in cancer research for the past several years [22], this review focuses on relatively lower abundance RNAs, such as yRNAs, tRF, tiRNA, snoRNA, snRNA, vRNA and piRNA [21]. These rare RNAs are highly conserved across species, play a role in physiological processes and are packaged in exosomes, making them potential exosome-associated diagnostic targets.

This is the era of personalized cancer treatment [7], in which significant emphasis is laid upon understanding individuals’ genetics, as well as epigenetic make-up, for effective therapy and prognosis, and exosomes can fit in as personalized diagnostic or prognostic biomarkers [23]. The exciting work to further our understanding of the precise role of exosomes in cancer progression remains in progress, and it is for this reason that a second volume of this Special Issue, ‘*Exosomes in Cancer Diagnosis and Therapy 2.0*’, is currently open for submissions. Given the promising results achieved so far, the progress of exosomes in cancer diagnosis, therapy and prognosis will be worth watching for the next several years.

## Abbreviations

circRNA	Circular RNA
DC	Dendritic cells
lncRNA	Long non-coding RNA
PD-L1	Programmed cell death ligand-1
piRNA	Piwi-interacting RNA
miRNA	MicroRNA
snoRNA	Small nucleolar RNA
snRNA	Small nuclear RNA
TNBC	Triple negative breast cancer
tiRNA	tRNA halves
tRF	Transfer RNA fragments
tRNA	Transfer RNA
vRNA	Vault RNA
